# Willingness to Participate in a National Precision Medicine Cohort: Attitudes of Chronic Kidney Disease Patients at a Cleveland Public Hospital

**DOI:** 10.3390/jpm8030021

**Published:** 2018-06-26

**Authors:** Jessica N. Cooke Bailey, Dana C. Crawford, Aaron Goldenberg, Anne Slaven, Julie Pencak, Marleen Schachere, William S. Bush, John R. Sedor, John F. O’Toole

**Affiliations:** 1 Department of Population and Quantitative Health Sciences, Case Western Reserve University, Cleveland, OH 44106, USA; dana.crawford@case.edu (D.C.C.); wsb36@case.edu (W.S.B.); 2 Institute for Computational Biology, Case Western Reserve University, Cleveland, OH 44106, USA; 3 Department of Bioethics, Case Western Reserve University, Cleveland, OH 44106, USA; ajg10@case.edu; 4 Division of Nephrology, Department of Medicine, MetroHealth Medical Center, Cleveland, OH 44109, USA; aslaven@metrohealth.org (A.S.); jpencak@metrohealth.org (J.P.); mschachere@metrohealth.org (M.S.); 5 Department of Medicine, Case Western Reserve University, Cleveland, OH 44106, USA; jrs4@case.edu (J.R.S.); jxo69@case.edu (J.F.O.); 6 Department of Physiology and Biophysics, Case Western Reserve University, Cleveland, OH 44106, USA; 7 Glickman Urological and Kidney Institute, Cleveland Clinic, Cleveland, OH 44195, USA

**Keywords:** biorepository, precision medicine, research participation, return of research results

## Abstract

Multiple ongoing, government-funded national efforts longitudinally collect health data and biospecimens for precision medicine research with ascertainment strategies increasingly emphasizing underrepresented groups in biomedical research. We surveyed chronic kidney disease patients from an academic, public integrated tertiary care system in Cleveland, Ohio, to examine local attitudes toward participation in large-scale government-funded studies. Responses (*n* = 103) indicate the majority (71%) would participate in a hypothetical national precision medicine cohort and were willing to send biospecimens to a national repository and share de-identified data, but <50% of respondents were willing to install a phone app to track personal data. The majority of participants (62%) indicated that return of research results was very important, and the majority (54%) also wanted all of their research-collected health and genetic data returned. Response patterns did not differ by race/ethnicity. Overall, we found high willingness to participate among this Cleveland patient population already participating in a local genetic study. These data suggest that despite common perceptions, subjects from communities traditionally underrepresented in genetic research will participate and agree to store samples and health data in repositories. Furthermore, most participants want return of research results, which will require a plan to provide these data in a secure, accessible, and understandable manner.

## 1. Introduction

Precision medicine research requires access to extensive health, lifestyle/behavioral, and genomic data on hundreds of thousands to millions of participants or patients. For many large studies such as the longitudinal Precision Medicine Initiative Cohort Program (now known as All of Us) [1,2] and the Million Veteran Program [3], participation includes periodic questionnaires, surveys, and physical exams supplemented by digital downloads of electronic health record (EHR) data and wearable technologies. Participation also typically includes submission of biospecimens, which may be sent to a central biobanking facility, possibly funded by the government, for storage and further processing for data generation [4]. Combined, these research data capture past and current health status as well as the potential risk factors that contribute to disease outcomes. 

Today’s comprehensive cohort studies expect continuous access to private health and exposure data linked to genomic data and stored as individual-level research data in large databases or cloud environments for analysis by multiple investigators [5]. For the individual investigator, this centralized model of biobanking, data storage, and data analysis is cost-effective and provides security, flexibility, and standardized quality control compared with a federated model wherein individual institutions would maintain physical storage of biospecimen and data collections [5,6]. For the individual participant, however, it is not clear how this centralized biospecimen and data storage model impacts their willingness to contribute to these large cohorts, particularly for those cohorts ascertaining participants from diverse socio-economic, geographic, and racial communities.

Also unclear are participants’ desires for return of research results and the type of research results they would like to receive. Return of aggregate research results is generally accepted by both researchers and participants, albeit under-utilized, for epidemiologic and biomedical studies, with individual-level research results returned only if clinically actionable [7,8,9]. Calls for the return of individual-level genetic research results have risen in the past decade and a half [10], coincident with the rise in biobank-based genomic research and direct-to-consumer genetic testing. To date, many existing genetic studies do not return individual-level research results [11], yet recent studies suggest that research participants are interested in receiving these results. Consequently, newer national efforts such as All of Us are considering return of individual-level research results, where format and frequency may be determined by the participant. 

Given the rise of larger longitudinal cohort studies such as All of Us, it is crucial to better understand the potential impact that centralized data storage and sharing models, as well as approaches to returning research results, may have on recruitment and retention of socioeconomically and racially/ethnically diverse participant populations. To that end, we surveyed patients in Cleveland, Ohio participating in a precision medicine research project at a large public health care system enriched in minority and low socioeconomic patients. In this diverse setting, we describe patients’ willingness to participate in precision medicine research including biospecimen collection, data sharing, and return of research results. 

## 2. Methods

### 2.1. Study Population

All study participants were ascertained at MetroHealth Medical Center. The MetroHealth System is an academic, public provider integrated tertiary care system serving Cleveland and northeast Ohio with a vendor-based (Care Everywhere, Epic Systems Corporation, Verona, WI, USA) EHR. Annually, the system has approximately 1.2 million outpatient visits, >100,000 emergency department visits, and almost 30,000 inpatient admissions, and the payer mix as of 2015 includes ~50% Medicaid, 25% Medicare, <25% commercial insurance, and 5% self-pay [12]. Almost 34% of residents near MetroHealth Medical Center’s main campus (zip code 44109) live below the poverty line, and at least eight of the 10 zip codes served by MetroHealth and highlighted in its community health needs assessment have higher rates of poverty (ranging from 21.1% (zip code 44111) to 56.1% (zip code 44104)) compared with Cuyahoga county as a whole (18.5%) [13,14]. The MetroHealth System is the safety-net hospital of the Cleveland area.

As part of a pilot study on precision medicine research in Cleveland, Ohio, we ascertained and consented patients followed in the MetroHealth System’s main campus nephrology clinics between March 2016 and July 2017 for biospecimen collection to be used in future kidney-related genomic research in diverse patient populations linked to EHRs. Pilot study participants consented to have their biospecimens stored locally and their EHRs accessible for research. Pilot study participants also consented to storing their de-identified research data and biospecimens in National Institutes of Health (NIH)-approved repositories and to data sharing outside of the pilot study. Eligible participants were asked by their physician during a routine clinic visit if they would participate in the Cleveland pilot study; those who agreed to participate completed formal in-person consent with a study coordinator and signed informed consent documents after all participant’s questions were answered regarding study participation. Study participants were assigned a study ID at the time of blood draw. We also asked participants to take a short, structured survey about their willingness to participate in a national precision medicine project. Surveys were then administered by the study coordinator. All procedures and processes including the survey were reviewed and approved by the MetroHealth System Institutional Review Board (Protocol ID: IRB13-00462). 

The survey was drafted by a single author (a nephrologist) followed by review and revision by four co-investigators (one nephrologist and three investigators in precision medicine research). The final draft was reviewed by a bioethicist and piloted among the authors before it was administered to participants. The survey was administered in written format with a Flesch Reading Ease of 41 at Flesch-Kincaid Grade Level 12, and data were transcribed manually for downstream analyses. The survey consisted of five questions:

1. Would you be willing to allow your health records and genetic information to be stored in a national biorepository coordinated by the government as a part of their “Precision Medicine Initiative”? If so this information may be available to researchers nationally and internationally with the understanding that your privacy would be protected.
2. Would you be willing to install a free phone app that would be able to track your physical activity, measures of your health and location with the understanding that your privacy would be protected?
3. Would you be willing to allow the information collected using the phone app (question 2) to be sent to the national coordinating center where it may be shared with researchers nationally and internationally with the understanding that your privacy would be protected?
4. If you participated in a study that collected your genetic and health information, how important is it to you that you receive results from the study? Circle the number that is closest to how you feel.
5. What type of results would you like to receive, please check all that apply:

Questions 1 through 3 allowed binary “yes/no” responses. Question 4 responses were allowed on a 1 to 5 Likert-type scale with 1 representing “not important at all” and 5 representing “very important”. For question 5, participants had the option of selecting as many scenarios as desired, which included (a) Information about the average results of all participants and nothing about yourself specifically; (b) Information about yourself that your doctor may have already provided to you (for example, smoking or excess weight), which may increase your risk for future health problems; (c) Information about your genes that may influence your doctor’s approach to your care (for example, they may order additional testing or consider alternative treatments or medications); (d) Information about your genes that has uncertain significance and will not change the way that your doctor treats you; and (e) I do not want to receive any results. All questions and possible responses were available to the participant at the time the survey was administered, and participants were free to leave blank any question they chose not to answer.

A total of 138 patients approached agreed to the blood draw for pilot study biospecimen collection and future genomics and/or filled out the survey. Of these, four surveys were excluded from further analysis because they were not assigned a study ID for the following reasons: one participant refused to donate a biospecimen but filled out the survey, one participant filled out the survey and agreed to donate a biospecimen, but blood could not be drawn, and two participants filled out the survey and donated a biospecimen, but the surveys were not assigned a study ID at the time of ascertainment. Of the remaining 134 participants, 21 donated a biospecimen but refused to or did not take the survey, leaving 113 surveys available for further analysis. 

### 2.2. Statistical Analysis

Survey readability and corresponding number of years of education required to comprehend it were assessed using the Flesch Reading Ease formula and the Flesch-Kincaid Grade Level Index [15,16,17], respectively, implemented in Microsoft^®^ Word 2016 (Microsoft, Redmond, WA, USA). Race/ethnicity, age at survey, and sex were extracted from the MetroHealth EHR, and subsequent analyses were limited to participants with these complete data (*n* = 103). Statistical analyses were performed in R. Chi-square tests (or Fishers Exact tests when cell counts were <10) were used to compare yes/no answers across race and sex. Race/ethnicity was extracted from the EHR clinical notes and likely represents a mixture of administratively-assigned and self-reported race/ethnicity. We further categorized race/ethnicity using US Census categories: White, Black or African American, American Indian and Alaska Native, Asian, Native Hawaiian and Other Pacific Islander, and Hispanic or Latino [18]. Comparisons between racial/ethnic groups were limited to Blacks or African Americans and Whites, the two largest groups.

## 3. Results

Of the 134 participants who agreed to donate a biospecimen for the local pilot study, a total of 103 (76.87%) with EHR-extractable demographic data completed the survey (Table 1). The average age of survey respondents was 61.45 (standard deviation = 13.52) years, and a majority of respondents were female (54.4%). Survey respondents were racially/ethnically diverse, representing Blacks or African Americans (50.5%), Whites (45.6%), Hispanics or Latinos (0.97%), Asians (0.97%), and American Indians or Alaska Natives (1.94%). A minority (21/134) of participants donated biospecimens but did not complete the survey. Of these, 12 had EHR-extractable data. Like those who took the survey, this group of participants was 50% Black or African American and 41.67% female with a mean age of 67.33 years. 

The short survey probed participants for opinions related to two general topics: participation in a national precision medicine research project (Table 2) and return of research results. The majority of participants (71.29%) indicated that they would be willing to participate in a national biorepository where their EHRs and genetic information would be stored (Q1). In contrast, only 44.66% (46/103) would be willing to install a free phone app to track lifestyle, behaviors, and environmental exposures (Q2). Three participants indicated they were not willing to install a free phone app, but they did indicate that they would be willing to send these app-collected data to a national coordinating center (Q2). Consequently, slightly more participants (47/97 or 48.45%) responded “yes” to Q3 compared with Q2. No statistically significant differences were observed in responses to these three survey questions when stratified by race/ethnicity.

**Figure 1:**
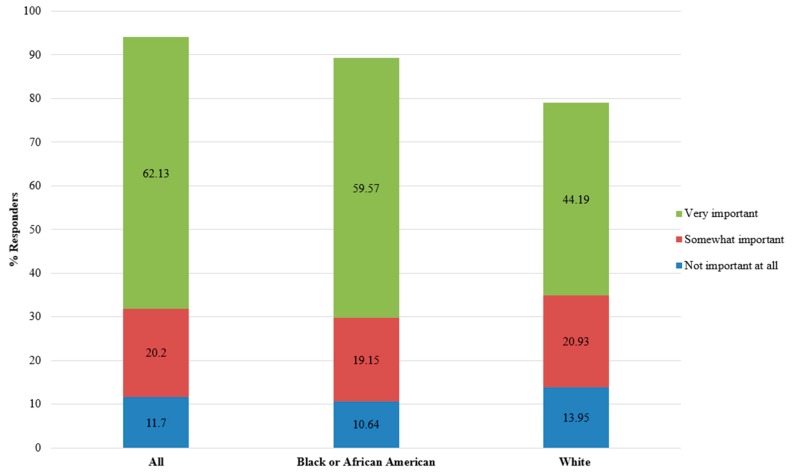
Survey question assessing the importance of return of genetic and health information. Survey participants (*n* = 101) were asked, “If you participated in a study that collected your genetic and health information, how important is it to you that you receive results from the study? Circle the number that is closest to how you feel.” A 1–5 Likert-type scale was used, where 1 represented “Not important at all,” 5 represented “Very important,” and the remaining values represented varying degrees of “Somewhat important”. Two respondents circled two numbers instead of a single number on the scale, and these ranks were then averaged (resulting in ranks 3.5 and 4.5). Three responses (not important at all, somewhat important, and very important) were plotted using a stacked chart for all responders (*n* = 94), Black or African American responders (*n* = 47), and White responders (*n* = 43; *x*-axis). The stacked chart is presented as % responders (*y*-axis) color coded by rank starting with rank 1 (“Not important at all” in blue) and ending with rank 5 (“Very important” in green).

The remaining two questions probed interest in return of research results. The majority of respondents (62.13%) indicated that it was very important to receive genetic and health results from the study (option 5; Q4), whereas only a small percentage (11.70%) of respondents indicated that return of study results was not important at all (option 1 for Q4; Figure 1). There was no statistically significant difference in responses when stratified by race/ethnicity, sex, or race/ethnicity and sex.

Participants were also asked to indicate what kind of results they would like to receive (Q5). Of those who responded to this question (94/103), only 10.64% (10/94) of respondents indicated that they did not want any research results returned to them (response e; Table 3). In contrast, 54.26% of respondents indicated that they were interested in all their genetic and health data. Summary-level data was of less interest to this patient population, with only 37.23% (35/94) of respondents selecting this choice in combination with other choices. There was no statistically significant difference in responses when stratified by race/ethnicity, sex, or race/ethnicity and sex.

We further characterized respondents who were interested in receiving research results (84/94; Table 4). Very few respondents only wanted summary-level data (only option a; 4.76% or 4/84) or only wanted health data (only option b; 3.57% or 3/84), whereas 20.02% (17/84) of respondents indicated that they wanted only genetic data (option c, d and c or d). The vast majority (80/84 or 95.24%) wanted data about themselves (options b, c, or d), including at least health data (option b at all; 63/84 or 73.81%), and at least genetic data (option c or d at all; 90.48% or 76/84). Again, there was no statistically significant difference in responses when stratified by race/ethnicity, sex, or race/ethnicity and sex.

A minority of participants (10/134) donated a biospecimen and completed the survey but did not have complete demographic data. The response patterns for these ten participants were similar to those with demographic data: 67% indicated that they would allow their health records and genetic information to be stored in a national biorepository, 56% would install a phone app, and 56% would allow data from the phone app to be sent to the national coordinating center. Furthermore, 50% of these participants indicated that it was very important to receive results from the study while two participants indicated that this was not important at all. Half of these participants wanted all data available returned to them, including study summary-level data, health data about themselves, and genetic data. 

## 4. Discussion

Cohort diversity is now a major focus for general translational genomics research [19,20,21,22] and consequently is a major goal for national precision medicine recruitment efforts [22,23]. Previous surveys and participation rate assessments for large, national genetic and genomic research initiatives, however, have suggested mixed reactions from different groups towards national research efforts that include collection of detailed health and lifestyle data and centralized biobanking [24,25,26,27,28,29,30]. In contrast, focus groups and surveys fielded between 2007 and 2015 have suggested that respondents regardless of race/ethnicity are more uniformly enthusiastic at the prospect of return of research results [28,29,31]. Here, we evaluated chronic kidney disease (CKD) patient attitudes towards national precision medicine research efforts via a short survey administered to individuals who had agreed to give a blood sample for local studies. This survey, fielded between 2016 and 2017, evaluated willingness to participate in different aspects of proposed national studies as well as importance of return of research results among patients in a CKD clinic at an urban public health care system.

Overall, we found that the majority (71%) of local research participants surveyed would be willing to have their EHR and genetic information stored in a national biorepository. The Cleveland area respondents were on average more enthusiastic compared with respondents of a recent national survey of whom 54% were willing to participate in hypothetical national precision medicine program and biobank [29]. This high level of hypothetical participation in a national research cohort might be expected given that all Cleveland survey respondents had already agreed to participate in our local study, which similar to the hypothetical national precision medicine program includes possible government (NIH) storage of biospecimens and data sharing outside the local study. Also, all participants are patients of the nephrology clinic, and their patient status as well as their relationship with the nephrologist may be major motivating factors in participating in biomedical research. While we did not collect data describing motivations for hypothetical participation in the national study, other surveys suggest that family history or personal experience with disease as well as patient-physician relationship are factors associated with participating in research that involves biobanking or genetic testing [32,33]. It is interesting to note that even though all Cleveland participants are CKD patients and are participating in our local study, 29% indicated they would *not* participate in a national study coordinated by the government, a response that did not differ by race/ethnicity.

Somewhat unexpectedly, 25% more participants were willing to allow health records and genetic information to be stored and available to researchers than were willing to install a phone app to track physical activity, measures of health, and location. These data suggest while a participant has potential excitement or indicates willingness to contribute to science, the extent of the actual contribution may have limitations. The differing levels of hypothetical participation may be due to several factors, including perceived response burden, concerns for privacy, or barriers to active participation (e.g., limited digital literacy). This survey did not probe for reasons of non-participation. However, given that select non-participation has serious consequences on longitudinal cohort studies where detailed and continuous data collection is expected, any initiatives to develop new cohort studies need to consider participation burden when designing best practices for consent and retention.

In assessing the importance of the return of research-collected genetic and health information, the majority of Cleveland-area respondents (76%) indicated that this was important or very important compared with 90% of national survey respondents [29]. Black or African American Cleveland-area participants placed more importance on return of research results (70.21%) compared with Whites (60.47%), although this difference was not statistically different. We did not evaluate how the participants interpreted the question “How important is it to you that you receive results from the study?” (Q4), and it is possible that some participants interpreted “health information” as information collected during a clinic visit and not as data collected as a part of a research study. While this distinction is important, the Cleveland-area response patterns were similar to other published surveys, suggesting at least similar interpretation or understanding of the general question regarding the perceived importance of return of research results. For example, while overall lower than the national survey, the Cleveland-area respondents were similarly interested in return of research results compared with a survey of veterans where ~67% of respondents indicated that they would not participate in a Veteran’s Affairs (VA) biobank if research results were not returned [27]. Compared with both the national survey and the VA survey, the present study has a higher proportion of Black or African American participants (50.5%) and older participants (61% ≥ 60 years of age). Although not measured at the individual-level in this survey, the overall MetroHealth System patient population from which this participant sample is drawn likely also has a higher proportion of low-income patients compared with the national survey [29]. Our survey also did not probe motivations for return of research results nor did we survey participants on their preferences in how results should be returned. Further data are needed to characterize and understand the impact of socioeconomic status has on the demand for return of research results as well as the media and means in which they should be returned. 

Interestingly, of the participants who indicated that they were interested in the return of research results, the majority who wanted data on themselves wanted at least genetic data as compared with at least health data. Participants’ rationale for distinguishing between research-collected health and genetic data is not clear, as it was not probed. Also not clear is why genetic data were more highly valued compared with research-collected individual-level health data. The general population overall is more aware of genetic testing, particularly with the advent of direct-to-consumer genetic testing [34]. Recent data suggest African Americans are very interested in genetic ancestry and traits, whereas Whites are very interested in drug response and genetic ancestry [35]. The present study did not suggest major differences by race/ethnicity, but further study is needed to better understand why participants in general value research-collected genetic data more than health data and if these preferences differ by age, race/ethnicity, and socioeconomic status, among other factors. 

The current study has several strengths and limitations. As already mentioned, one limitation is sample size. While our participant population included individuals from five race/ethnicity categories (White, Black or African American, American Indian and Alaska Native, Asian, Native Hawaiian and Other Pacific Islander, and Hispanic or Latino), sample sizes were only large enough in the White (45.6%) and Black or African American (50.5%) sample categories to make meaningful comparisons between groups. Approximately half of participants were female (54%). Comparing survey responses of Black or African American and White individuals, regardless of sex, did not highlight large differences in response patterns, consistent with findings from the national survey [29]. Statistical power, however, was limited in detecting smaller differences in response patterns.

Another limitation is possible misinterpretation or comprehension of the questions. Previous surveys suggest lower education level and increased age, among other factors, are associated with lower comprehension of biobanking consent forms and follow-up surveys [36]. This study population ranges in age from 18 to 91, with an average age of 65 years. Although not explicitly tested, it is possible that the older participants had less survey comprehension or more misinterpretation compared with younger participants in this and other surveys. It is also possible that this population has less education compared with other populations surveyed for participation in precision medicine research. While education level was not directly assessed in this study, the American Community Survey 2016 [37] reports that 81% of Clevelanders graduated high school and only ~16% of Clevelanders have a Bachelor’s degree or higher, whereas 90% and 32% of the participants from the national survey completed high school and a Bachelor’s degree, respectively [29]. The present survey has a Flesch-Kincaid Grade Level 12, which may be too high for participants who have not completed high school. Interestingly, lower education in other surveys is also associated with lower willingness to participate [30], a result not evident in this study sample.

Survey respondents were limited to CKD patients participating in a genetic study; consequently, attitudes from non-respondents are not available. Consistent with a survey of biobank participants [38], few respondents indicated that return of research results was not important. The lack of observed differences between White and Black or African American responses in this survey of CKD patients is consistent with the national survey [29], as well as a survey of dialysis patient family members [39]. Among 130 first-degree relatives of patients on dialysis, an overwhelming majority of family members surveyed indicated that they wanted genetic results regardless of available treatments [39]. Similar to our report, approximately half of respondents were African American and half were European American, and attitudes regarding genetic testing results did not significantly differ between racial groups in their study [39].

Despite these limitations, we find that the majority of participants surveyed, regardless of race/ethnicity, are interested in participating in national precision medicine research cohorts or a similar study and that return of research results is important to them. These data suggest that the research community should be prepared to provide research results, and further studies are needed that engage the public and patients to determine the optimal delivery methods and mechanisms to ensure that understandable information is disseminated responsibly to all who request it.

## Figures and Tables

**Table 1:** Survey respondent demographics. Race/ethnicity, age at survey, and sex were extracted from the MetroHealth System electronic health record for consented patients who provided a biospecimen and completed the survey. Other includes American Indian and Alaska Native, Hispanic or Latino, and Asian.

	Overall	Black or African American	White	Other
**Samples size**	103	52	47	4
**% Female**	54.40	57.69	51.06	50.00
**Mean age, in years (SD)**	61.45 (13.52)	61.42 (13.02)	62.38 (13.37)	50.75 (15.42)

Abbreviations: standard deviation (SD).

**Table 2:** Survey questions assessing willingness to participate in the Precision Medicine Initiative Cohort Program. A total of 103 consented patients who donated a biospecimen completed the survey. Not all questions were answered by survey participants; therefore, the denominator varies by question and group.

1. Would you be willing to allow your health records and genetic information to be stored in a national biorepository coordinated by the government as a part of their “Precision Medicine Initiative”? If so this information may be available to researchers nationally and internationally with the understanding that your privacy would be protected.			
**Response**	**All Respondents**	**Black or African American Respondents**	**White Respondents**
% Yes (*n*)	71.29 (72/101)	69.23 (36/52)	71.11 (32/45)
2. Would you be willing to install a free phone app that would be able to track your physical activity, measures of your health and location with the understanding that your privacy would be protected?			
**Response**	**All Respondents**	**Black or African American Respondents**	**White Respondents**
% Yes (*n*)	44.66 (46/103)	46.15 (24/52)	38.30 (18/47)
3. Would you be willing to allow the information collected using the phone app (question 2) to be sent to the national coordinating center where it may be shared with researchers nationally and internationally with the understanding that your privacy would be protected?			
**Response**	**All Respondents**	**Black or African American Respondents**	**White Respondents**
% Yes (*n*)	48.45 (47/97)	54.00 (27/50)	37.21 (16/43)

**Table 3:** Survey question assessing preference in return of research results. Of the 103 survey respondents, 94 answered this question. Respondents could select more than one category, which included (a) Information about the average results of all participants and nothing about yourself specifically; (b) Information about yourself that your doctor may have already provided to you (for example, smoking or excess weight), which may increase your risk for future health problems; (c) Information about your genes that may influence your doctor’s approach to your care (for example, they may order additional testing or consider alternative treatments or medications); (d) Information about your genes that has uncertain significance and will not change the way that your doctor treats you; and (e) I do not want to receive any results.

Answer Included at Least:	% All Respondents	% Black or African American Respondents	% White Respondents
a	37.23	28.84	36.17
b	67.02	59.62	59.57
c	76.60	71.15	68.09
d	61.70	57.69	53.19
e	10.64	9.62	10.64

**Table 4:** Return of research results preferences among respondents who want results. Of the 103 survey respondents, 94 answered this question. From this, we excluded respondents who did not want any results (*n* = 10). Respondents could select more than one category, which included (a) Information about the average results of all participants and nothing about yourself specifically; (b) Information about yourself that your doctor may have already provided to you (for example, smoking or excess weight), which may increase your risk for future health problems; (c) Information about your genes that may influence your doctor’s approach to your care (for example, they may order additional testing or consider alternative treatments or medications); (d) Information about your genes that may influence your doctor’s approach to your care (for example, they may order additional testing or consider alternative treatments or medications). From these categories, we further categorized preferences as (1) at least summary data (A at all), (2) only summary data (only A), (3) a least health data (B at all), (4) only health data (only B), (5) at least genetic data (C or D at all), (6) only genetic data (only C, D, or C and D), and (7) at least genetic and health data on self (B, C, or D at all).

Response Category	% All Respondents	% Black or African American Respondents	% White Respondents
At least summary data	40.48	33.33	44.74
Only summary data	4.76	2.38	7.89
At least health data	73.81	71.43	73.68
Only health data	3.57	2.38	2.63
At least genetic data	90.48	92.86	89.47
Only genetic data	20.24	26.19	15.79
At least data on self	95.24	97.62	92.11
